# Queer(y)ing aging—potentialities and problems in applying Queer Theory to studies of aging and later life

**DOI:** 10.3389/fsoc.2023.1228993

**Published:** 2023-09-29

**Authors:** Andrew King, Matthew Hall

**Affiliations:** Department of Sociology, University of Surrey, Guildford, United Kingdom

**Keywords:** LGBTQ, queer, theory, aging, life course

## Abstract

Queer Theory is a radically deconstructionist perspective within the humanities and social sciences. Since its initial emergence in the late 1980s and early 1990s in the field of sexualities studies, Queer Theory has increasingly been used to challenges normative notions of self, identity, temporality and the nature of being, more broadly. Whilst Queer Theory has been utilized, to some extent, in gerontology and aging studies, this article makes an original contribution to this endeavor, assessing the potentiality and problems with queer(y)ing three aspects of aging: chronology; cognition; and frailty and vulnerability. To achieve this, the article draws on ideas from some key Queer theorical writers, existing studies of queer aging and illustrates theoretical points with qualitative data collected from two LGBTQ+ projects to illustrate. The article also considers problems with Queer Theory in challenging normativities associated with aging. It is concluded that despite problems, Queer Theory remains an important and valuable theoretical approach for disturbing and challenging many of the norms and understandings that shape and constrain older LGBTQ+ people's lives, in particular, and therefore have importance for how we think and understand aging and later life sociologically.

## Introduction

Sociology has long considered ways that aging and later life are framed by social, economic, cultural, and political factors. Whilst there are statistics about general trends in population aging and how societies are becoming increasingly “aged,” much less has been written about why we need to approach aging and older people *differently*. By this, we do not mean in terms of popular concepts such as success, wellness or health, as are so often posited in scholarship, policy and practice. Instead, we mean the need to think about aging more critically, more sociologically, to challenge the norms that shape aging lives.

In our article, we want to further this more critical and challenging focus on how we understand aging by drawing on Queer Theory, a radically deconstructionist perspective within the humanities and social sciences, including sociology, which challenges binary logics and thinking (Green, 2007; McCann and Monaghan, 2020). Queer Theory emerged from academic scholarship associated with post-structuralism and post-modernism, alongside and in relation to activist debates associated with the AIDS epidemic of the 1980s. It also addressed growing dissatisfaction toward the mainstream lesbian and gay rights movement of the time.

Queer Theory is often considered to be synonymous with studies of lesbian, gay, bisexual, trans, queer, and other gender and sexually diverse (LGBTQ+) people – as a theory about gender identity and sexuality that challenges cisheteronormativity, the pervasive norm that everyone is cisgender and heterosexual unless indicated otherwise. Whilst this article does use Queer Theory in this way, since the studies and illustrative data examples we draw on do concern LGBTQ+ people's lives, our call to queer(y) aging has wider implications for thinking about normativity and later life, as we note in the discussion and conclusion. As such, our article contributes toward the growing but still limited scholarship that has applied Queer Theory to aging. Here our original contribution to the field is to show how Queer Theory can be utilized to critically engage with several different aspects of aging, to challenge the ways that normativity is manifested through them and to further sociological knowledge concerning the complex and often contradictory implications that this has for our understandings of later life. We do this by recourse to: chronology and a normative lifecourse; cognition, particularly normative ideas about cognitive capacity; and normative ideas about frailty and associated vulnerabilities. Overall, and despite some problems with Queer Theory itself, we will demonstrate its usefulness for enabling us to further understand aging from a sociological perspective.

The first section of the article provides a brief sketch of Queer Theory and its application to the sociology of aging, before the second section briefly outlines the methodologies of the two empirical projects we are drawing on in this article: “Comparing Intersectional Life Course Inequalities amongst LGBTQI+ citizens in four European Countries” (hereafter CILIA) which explored LGBTQ+ inequalities across the life course and “Secure, Accessible, Friendly, Equality Housing” (hereafter SAFE Housing), which explored the concerns, preferences and experiences of older LGBTQ+ people in relation to housing. In the third section, we interlace a theoretical discussion that applies Queer Theory to the three aspects of aging previously noted, with empirical examples i.e., relevant participant quotations taken from these two projects. The article is not a systematic thematic analysis of these findings; it is a theoretical discussion that uses empirical examples. The fourth section then draws the preceding analysis together, highlights some key issues with applying Queer Theory to aging, as well as some problems. In the discussion and conclusion we speculate how a queer sociology of aging could be further taken forward.

## Queer Theory—a theory without a subject?

Defining Queer Theory is difficult. It is not a unified body of work, nor does it have a singular subject at its core – be it an individual, a subject position or a disciplinary locus. McCann and Monaghan (2020) suggest, somewhat ironically, that its indefinability is its defining feature. Nevertheless, Queer Theory can be thought of as a corpus of often contradictory anti-foundational ideas and ways of viewing knowledge and subjectivity that have emerged over the past 30 years within the humanities and social sciences, including sociology (Green, 2007). Whilst Queer Theory has generally been applied to the study of gender and sexualities, it is by no means confined to those topics.

Queer Theory, as a term, may usually be regarded as a noun that seeks to describe a conceptual perspective, but it also incorporates the notion of queering, or *to queer*, an active verb. Sullivan (2003, p. vi) suggests that to queer is “to make strange, to frustrate, to counteract, to delegitimize, to camp up (heteronormative) knowledge and institutions”. In contrast, Edelman (2004, p. 17) suggests queering “can never define an identity; it can only ever disturb one.” Drawing on these ideas, we utilize Queer Theory and the idea of queering somewhat simultaneously and interchangeably in this article to refer to a theoretical and conceptual set of ideas that challenge, trouble and disrupt anything that it is applied to or attempts to understand: in this case, aging.

Whilst Queer Theory cannot be said to have a unifying core, queer scholarship generally seeks to denaturalize identities and subject positions, arguing instead that these are emergent and performative in relation to historically specific discourses: for instance, both gender and sexuality, as properties of self, are ontological positions that emerged in Western societies from the scientific and industrial revolutions of the eighteenth and nineteenth Centuries (Foucault, 1978; Butler, 2004b). In this respect, to take up the subject position of a person, to have personhood, is to be positioned by and within available gendered and sexualized typologies, which include: man, woman, heterosexual, lesbian, gay, bisexual etc. Furthermore, the act of naming oneself, or being named by others in relation to these subject positions, is not typically agentic – as Butler (1993, p. 232) notes in relation to gender:

“(femininity) is thus not the product of choice, but the forcible citation of a norm, one whose complex historicity is indissociable from relations of discipline, regulation, punishment. Indeed, there is no “one” who takes on a gender norm. On the contrary, this citation of the gender norm is necessary in order to qualify as a “one,” to become viable as a “one,” where subject-formation is dependent on the prior operation of legitimating gender norms.”

Nor is this process fully circumscribed—in taking on such a subject position, or being interpolated into it, one does it *differently* since there is no authentic origin from which such subject positions are based. In short, within a performative norm there is the potential for its subversion. We will come back to some of these inherently deconstructive tendencies within Queer Theory later in the article when we consider how this might apply to aging.

Queer Theory is often read as synonymous with LGBTQ+ lives and with its potential to deconstruct normativities associated with reproductive heterosexuality and cisgenderism. It has been used widely to illustrate the power of these normativities within cultures to shape and constrain lives (McCann and Monaghan, 2020). However, there are trends within Queer Theory which are pertinent for challenging existing understandings of identity and subjectivity beyond a conceptual focus on gender and sexuality *per se*.

Queer Theory is only one critical theory than can be used to critique norms around gender identity and sexuality across the life course – others could include Lahire (2011) concept of the plural actor, ethnomethodology (West and Zimmerman, 1987) and above all intersectionality (Taylor et al., 2010). However, there is an emerging corpus of work within disciplines allied to sociology, such as social work, social gerontology and public health, which have begun to apply Queer Theory to aging and it is that approach, above all, that we wish to extend in this article.

Sandberg and Marshall (2017) utilize Queer Theory alongside crip and feminist theories to critique notions of futurity incorporated into ideas about successful, positive aging which are used extensively in gerontology. Meanwhile, in a detailed qualitative study of older trans people, Fabbre (2015), also critiques a cisnormative view of successful aging and argues for the reconceptualization of “failure” and “success” to take account of different experiences rather than normative ones. Similarly, Ramirez-Valles (2016) argues that the study of aging needs to be queered, to avoid a universalistic, objectivist perspective that above all re-inscribes heteronormativity. In a summation of the value of queering gerontology Sandberg and King (2019) argued that applying Queer Theory to aging can highlight the effects of heteronormativity to shape and constrain aging lives and critique notions of chronology and the life course. Yet as is often the case with Queer Theory, what is also needed is a more grounded sociological analysis to fully evaluate its usefulness, or otherwise (King and Cronin, 2010; McCann, 2016). In this article, this means taking different aspects associated with aging and critically engaging with them with queer theoretical ideas and concepts to further develop and extend a queer sociology of aging. To do this, we will draw on Queer Theory to *queer* three aspects of aging to highlight the norms that frame them and how individual LGBTQ+ people negotiate and/or reproduce them– chronology, cognition and frailty/vulnerability. Before moving on to undertake this predominantly theoretical exposition utilizing empirical examples, we offer a methodological sketch of the two projects we are using for this purpose, which is prefaced by some details about the legal, political and cultural context related to LGBTQ+ lives in England, the location of the data on which we will draw. We are mindful that theoretical expositions, such that we are undertaking here, can and do look different in other geopolitical contexts and we are not offering a universal theory. Instead, we want to highlight theoretical notions that may be useful for others to further extend in their own and other contexts.

## Materials and methods

A variety of surveys have attempted to estimate the size of the LGBTQ+ population in England. Both gender identity and sexual orientation questions were included on the 2021 Census, a survey of all people and households in England and Wales undertaken every ten years. This indicated that 0.5% of the population of England and Wales over the age of 16 defined their gender identity as different from that assigned to them at birth. Meanwhile, 3.2% defined their sexual orientation as LGB+, 0.03% identified as queer. Both gender identity and sexual orientation questions were voluntary and arguably under-represent the real number of LGBTQ+ people in England.

Whatever the exact number of people in England who are LGBTQ+, there has been considerable legislative change related to gender identity and sexual orientation over the past fifty years. This has shifted, often unevenly and not without forms of backlash, from outright criminalization to more benign tolerance and subsequently more acceptance and protection. This process commenced with the partial decriminalization of male homosexual acts in England and Wales in 1967. Lesbian relationships were never criminalized, but women were subjected to discriminatory practices in terms of gender in relation to pay, pensions, and child support (Traies, 2016). During the late 1980s, Section 28 of the Education Act was introduced that explicitly prohibited the intentional “promotion” of homosexuality by local government authorities. This was repealed in 2003. Indeed, since the early 2000s a raft of legislation has sought to provide protection from discrimination in the provision of goods and services and in the Equality Act (2010) both gender reassignment and sexual orientation were classified as protected characteristics. Other important legislation has included: the Gender Recognition Act (2004) which enabled trans people to gain a gender recognition certificate to legally change their gender; whilst the Civil Partnership Act (2004) and the Marriage (Same-Sex Couples) Act (2013) have been important milestones toward equality in partnership and relationship recognition.

Simultaneously, the social and cultural context has shifted in England. The British Social Attitudes survey, a representative panel study that has been conducted annually since 1983 has documented this transformation. The number of responses to the suggestion that relations between people of the same-sex are “not wrong at all” has moved from 11% in 1987, up to 39% in 2007, 47% in 2012, and 68% in 2017. However, views on trans rights have been more mixed, as a comprehensive survey conducted by the polling organization YouGuv indicated in 2022 (Smith, 2022). This demonstrated that support for transitioning, trans rights and legal protection was greater amongst younger groups, those with higher educational qualifications and dependent on questions about context e.g., public toilets, sports, access to spaces.

It is against this legislative, social and cultural backdrop that current generations of LGBTQ+ people have lived their lives. Older LGBTQ+ people have experienced an earlier life where discrimination and hostility were much more common, and this has had an impact on how they experience later life (King, 2016a; Westwood, 2016a). Younger people, whilst living their lives in an apparently more inclusive culture, nonetheless experience issues that impact their wellbeing and mental health (McDermott et al., 2017). Moreover, it is important to remember that LGBTQ+ communities are diverse and incorporate individuals with multiple experiences and differences (Formby, 2017). Again, we want to emphasize that we are not offering this article as a homogenous, universalized (queer) theory of aging, but a more critical engagement with three aspects of aging using Queer Theory. Therefore, to illustrate this, we draw on data from two projects, which we outline below to give the reader an understanding of what they were about and how they related to aging.

Between 2018 and 2021 we conducted a multi-partner study which explored life course inequalities experienced by LGBTQI+ people in four European countries: England, Germany, Portugal and Scotland (CILIA). The CILIA project comprised four work-packages, conducted in each country: (1) a discourse analysis of legal and policy documents selected from each country; (2) secondary analysis of survey data related to LGBTQ+ people in Europe; (3) qualitative interviews with LGBTQI+ adults undertaken by research teams in each country that explored inequalities across their lives; and (4) agent-based modeling of workplace inequalities that synthesized data from the three other work-packages.

In this article we will draw on the 48 qualitative interviews we conducted in England, as we have full access to this data^1^ and it fits with the scope of this article to illustrate our theoretical ideas. Along with another researcher, we interviewed 48 LGBTQI+ people in England aged between 18 and 84 years of age although the majority were aged between 40 and 60 years of age, i.e., those years normatively classified as middle-aged. In terms of sexuality, 12 participants defined their sexuality as lesbian/gay women, 12 as gay men, 8 bisexual, 10 queer, 1 pansexual and 3 as queer bisexual. Regarding gender and gender identity, 20 defined themselves as women including 2 transwomen, 20 as men, including 3 transmen and 8 as non-binary, genderqueer or gender diverse. 41 participants identified as White and 7 identified as having a Black, Asian or minority ethnicity. Nearly half of the participants (*n* = 23) declared a long-term health condition and a third of participants (*n* = 15) declared a disability. In terms of relationship status and living arrangements, 18 self-identified as single, 4 as married or in a civil partnership, 2 as widowed, 6 living with a partner, whilst the remaining 18 mentioned various forms of monogamous or open/polyamorous partnerships. In terms of class background, 18 self-identified as middle-class, 3 lower-middle-class, 1 between middle-class and working class, 1 upper-working class, 24 as working-class and 1 gave no comment.

Interviews were semi-structured around a topic guide that included questions about: early life and school, family (of origin and choice), experiences in the workplace, experiences of discrimination, revealing one's gender identity, sexual orientation or intersex status to others, issues around later life and retirement and perceptions of LGBTQI+ equalities. All were encouraged to talk about their earlier life experiences but also to consider what aging as a LGBTQI+ person may be like. Interviews lasted on average 90–120 min. To ensure ethical practice, the study was approved by the University of Surrey Ethics Committee and all participants received participant information sheets and consent forms in advance. Any concerns about confidentiality or anonymity were discussed with the participant before they consented and at the start of the interview. The interviews took place between March 2019 and May 2020. Even before the COVID-19 pandemic lockdowns commenced, the interviews were primarily conducted via online meeting platforms, to facilitate the geographical diversity of participants, although around a third were interviewed in person. Participants came from a wide range of areas of England, which covered rural, suburban and urban communities. All interviews were transcribed and a thematic analysis (Boyatzis, 1998; Seal, 2016) was conducted by the second author with coding checks on 10% of the sample by the lead author.

The Secure, Accessible, Friendly, Equal Housing (SAFE) project was an earlier study conducted by Andrew King and others, which focused on the housing experiences, concerns and preferences of LGBT+ people aged over fifty years of age. Undertaken in 2016 to address gaps in knowledge concerning older LGBT+ people and housing in later life, SAFE Housing utilized a mixed methods design. In addition to a survey, which garnered a total 175 usable responses, 26 participants took part in five focus groups in two different regions of England, which covered urban, suburban and rural areas. Focus groups were chosen because they can be used to facilitate the sharing of experiences and produce group level ideas about a topic (Cronin, 2008). In this case, focus groups were used to explore collective ideas about issues related to housing and what types of housing might be required in the future as LGBTQ+ people get older.

Amongst the qualitative sample, 13 participants identified as women and nine as men. Four people self-identified as trans. A total of 14 participants identified as lesbians, nine as gay men, one as a bisexual woman and two as pansexual. The majority of the focus group sample were aged 50–69 years (7%), although four people (15%) were aged 70–79 years and three people were aged 80–85 years (12%). Two-thirds of the sample were in some form of relationship/ relationships, whilst a third were single. A total of 73% of the sample were owner-occupiers. A total of 58% of the sample lived in the urban conurbation and 42% in the more rural county. All the focus group participants identified as “white British,” which we recognized was as a limitation in the research. The majority also defined themselves as middle-class. We did not specifically ask participants to identify if they had a health condition or disability. The project was approved by the University of Surrey Ethics committee.

Focus groups were conducted in person, led by a researcher and recorded. They lasted on average 120 min. The audio recordings were transcribed and a thematic analysis (Boyatzis, 1998; Seal, 2016) was undertaken. This produced three over-arching themes: safety, comfort and trust at home; community and connections; and future housing. However, within these core themes, subthemes related to experiences of aging, including those concerning chronology and norms associated with it, cognition and cognitive capacity, alongside vulnerability and frailty, did emerge despite the more collective, group setting of the focus group. Therefore, we have also used some of this data in this article to further illustrate the theoretical arguments which follow.

## Results: queer(y)ing aging in three ways

As we have been arguing in the preceding sections, Queer Theory's broad applicability is part of its appeal. As well as drawing on some canonical queer writings in this section we use examples from our own research projects to illustrate their more grounded aspects and to further develop and extend a sociological queer(y)ing of aging.

### Queering chronology

Aging can be conceptualized as the passing of time, whilst chronology the passing of time in linear and ordered ways. The importance of chronology in shaping and constraining lives has been the subject of some key texts within Queer Theory. Included here are ground-breaking writings by Halberstam (2005) and Freeman (2010), who both illustrate the deleterious effects of normative clock time and a normative life course on LGBTQ+ lives. These norms of chronology are further compounded by the existence of what Edelman (2004) refers to as reproductive futurity, a (hetero)normative life course predicated on the cultural valorization of the Child.

For Freeman (2010, p. 3) the emergence of industrial clock time in the nineteenth century led to the development of chrononormativity: “the use of time to organize individual human bodies toward maximum productivity.” In a highly rigid, capitalist context which separates productive bodies from unproductive ones, bodies that are deemed productive are valorized, those that are deemed unproductive are unviable, disposable. In this sense, normative chronologies shape and constrain lives, particularly those deemed queer by recourse to the unproductiveness or their failure to follow normative life course trajectories.

The questioning of a normative chronology across the life course is also something that is found in Halberstam's (2005) seminal “In a Queer Time and Place: Transgender Bodies, Subcultural Lives.” Arguing for the notion of “queer time” detached from normative chronologies of family, inheritance and reproduction, Halberstam equates “queer temporalities” with a messiness and stretched-outness of normative life course times and stages – for instance, taboos about acting your age and doing things in the right time and place and order. For Halberstam, LGBT+ lives, in particular, challenge these and create possibilities for new modes of living outside of normative chronological times. Indeed, in *The Queer Art of Failure* Halberstam (2011) looks at the potentiality of failing to adhere to such norms, particularly within queer kindship, as a way of highlighting both their persistence but also fragility.

Meanwhile Edelman's (2004) critique of reproductive futurity and the way that childhood is valorized symbolically within Western cultures indicates the inherent queerness of those whose life course does not fit within or conform to a normative, familial and reproductive time. Unable to reproduce in heteronormative ways, queers are “out of time,” associated with a negativity from which they cannot escape. Edelman argues that instead of viewing this as something which must be overcome, it is something that should be embraced.

It is clear from these different Queer writings that chronology, as it is practiced in many Western societies, such as England and increasingly globally, shapes and constrains all lives, but especially those who are deemed to be inherently unproductive or a challenge to a productive future. As others have indicated (Fabbre, 2015; Sandberg and King, 2019), this has important implications for aging as an LGBTQ+ person and how people have liveable (or indeed unviable) later lives (a phrase that is laden with symbolic norms about time!) and how aging is represented symbolically.

Most younger participants in the CILIA project had not thought much about aging and later life, but some had. For instance, one young cisgender lesbian (25–34 years age group), spoke about having a partner who was 20 years older than herself:

I don't know whether there'll be any challenges there, especially as she gets older and thinking forwards about caring for her and how people read our relationship, because they don't always necessarily realize that we're a couple at first. And so people might — they're not quite sure how to read it and she's even been mistaken for my mum previously. And I don't know whether that could happen as she's older. Who knows what might happen, I can just imagine if she's in hospital or something, that is something that I would worry about. We are planning to get married and I hope that that will alleviate that a little bit. Because then it gives you a bit of extra, I don't know, I'm like, “no, that's my wife come to me when things pass”

This younger lesbian's narrative highlights important points about aging within a relationship that challenges normative temporalities but also ways that individuals seek to mitigate the effects of structural constraints both in the present and in thinking about later life. Sociologically, it represents a queering of “how to read” non-normative aged relationships that transgress familial logics (i.e., they are not mother/daughter), but at the same time draws on changes in legislation such as the ones we highlighted earlier and equality laws to substantiate this. The use of the term “wife” following this participant's reference to “marriage” is a case in point. This younger lesbian makes it apparent that she requires this legal designation and validation to challenge a heteronormative temporality around next of kin. There is, then something both challenging (and troubling) to existing chronologies, but not without recourse to legislation.

For some of the older people who participated in the CILIA project, queer chronologies were evident in other ways, which also highlight both normative and non-normative aspects of aging. These narratives can be heard in the way that some participants sought to transform existing, heteronormative temporalities and create alternate narratives for themselves. The following example, from an older cisgender lesbian (65–74 year age group) who specifically highlights “worst” and “best” examples of her current life illustrates this:

Worst thing at the moment, getting old, things like. I've got cataracts developing, I need to watch the blood pressure and I'm never going to get my teenage years back again. Best thing about now, getting old and not giving a fuck and not having to work for a living. I volunteer here (LGBT+ organization), I'm on the management committee of a disability arts project and I dance. That's it and I have cats!

Whilst this older lesbian's narrative around bodily and attitudinal change could be heard widely amongst all older people, there does not seem to be much in the way of challenging norms of aging to be lamenting its effects on the body, it nevertheless has a *queer* inflection in terms of the progressive and the disruptive. There is a sense of “camping up” a narrative of decline and emphasizing the “not giving a fuck” agentic twists. Not all LGBTQ+ will do this, of course. This woman's professional background could well have been important. Indeed, despite these more agentic and positive narratives, some CILIA project participants narrated chronologies that reflected Edelman's notion of homonegativity – by embracing a more nihilistic and apathetic narrative, albeit one with a distinct temporal motion of its own. This is evident in the quote from a cisgender older gay man (75–84 year age group):

So when I'm at my lowest, I ask the question “will I still be alive this time next year?” But that I've learned how to put that on the back burner, and think in terms of how things will be now, perhaps tomorrow, possibly next week, probably a month or two. But after that, it's not so secure. Does that answer the question?

Indeed, in this respect, questioning a future by recourse to a more profound focus on the present, moving toward “no future” is inherently queer in the reproductive and familial logics of a chrononormative life course. What these differing narratives indicate for us is the way that LGBTQ+ people whatever their age (but including older LGBTQ+ people) often have to write their own narratives and scripts of aging, largely because as others have noted, older LGBT+ people were more hidden and silenced in the past, compared to the present (Pugh, 2002; Rosenfeld, 2002; Cronin, 2006). Nonetheless, futurity remains present in much aging research (Sandberg and Marshall, 2017) and a developing queer sociology of aging must recognize and critique it further.

### Queering cognitions

Where temporality has been deconstructed in Halberstam's Queer Theory, their work on forgetting is also very pertinent for challenging norms associated with cognition, or cognonormativity – the notion that there is a benchmark of cognition and anything which deviates from it is in some way pathological (Halberstam, 2011; King, 2022). Dementia is a case in point. Primarily a condition of later life, it is frequently represented in medicalized and catastrophic terms, as a failure in cognition caused by underlying biological and physiological changes. It is viewed as a global scourge with long-term impacts on individual lives, health care systems and overall population and societal wellbeing. For instance, the World Health Organization suggests:

dementia is currently the seventh leading cause of death among all diseases and one of the major causes of disability and dependency among older people worldwide. Dementia has physical, psychological, social and economic impacts, not only for people living with dementia, but also for their carers, families and society at large (World Health Organization, 2021).

There is a growing literature concerning the experiences of dementia amongst LGBTQ+ people, particularly in the UK, US and Australia. Two recent scoping reviews of 27 studies (Di Lorito et al., 2022; Smith et al., 2022) highlight several key issues and themes. These include: LGBT+ people having a heightened risk of developing dementia compared to cisheterosexual peers; experiences of dementia care are shaped by previous histories of oppression, especially with institutionalized forms of discrimination; concerns about a loss of a specific LGBTQ+ identity; LGBTQ+ social networks as mediators of resilience, providing much needed informal support; and LGBTQ+ carers are reluctant to seek support. In short, these scoping reviews, indicate that LGBTQ+ people are likely to have poorer outcomes in how they experience and are supported with dementia.

Whilst these reviews and the studies they are based upon are important, in this article we want to consider how Queer Theory can help us to understand dementia in different ways and contribute toward a queer(y) of aging. In “The Queer Art of Failure” Halberstam (2011) proposes the radical potential of forgetting by providing a close reading of the Disney film, “Finding Nemo.” Halberstam's cultural reading may appear to be much removed from the challenges of people living with dementia. However, through one of the protagonists anti-linear temporalities, forgetful diversions and non-familial forms of kinship, Halberstam offers a queer alternative to forms of ciscognonormativity (King, 2016b; Baril and Silverman, 2019) that provide important ideas for how dementia is understood and experienced by LGBTQ+ people. Indeed, we quoted a cisgender gay man in the previous section of this article as challenging temporal norms, but his forgetting of the future can also be considered a form of queer cognition, albeit one that is chosen from a standpoint of rationality i.e. he has chosen to forgo questions of longevity beyond a few months.

As one of us has argued elsewhere (King, 2022), dementia can be read as synonymous with queerness, regardless of the gender identity and sexual orientation of the individual. This is because those with dementia are often symbolically regarded as disruptive of a normative life course and successful aging process. The behaviors and narratives of those living with dementia are regarded as troublesome to cognitive normalcy and indicative of cognitive abnormality. Indeed, many organizations and individuals, including some LGBTQ+ individuals, attempt to reframe dementia as something that is containable within existing norms related to family and futurity, as something that, despite its deleterious impacts, can somehow be recuperated with care and understanding, within existing norms (King, 2022). Many studies of LGBTQ+ people's experiences of dementia seek to offer solutions that are largely containable within these framings including more focused, person-centered care (Bailey et al., 2022). In other words, LGBTQ+ people are as likely as cisgender heterosexual people to reproduce framings of dementia as “thief” or loss of self.

In the CILIA project interviews, dementia was discussed by several participants who had relatives or friends who had been diagnosed, or who had been involved in caring in voluntary or paid capacities for people with dementia. What was particularly striking in these participant narratives was: (a) how dementia was reinforced as a challenge to queerness, as a threat to LGBTQ+ identity; and/or (b) as something that a queer relational framing could support.

One middle-aged cisgender lesbian (45–54 year age group) spoke about how an older lesbian friend and her partner were heterosexualised by care providers:

She lived with her partner for about 35 years and her partner's got dementia and is in a care home and she contacted me saying, “I'm really struggling, do you know any groups, do you know anyone, any support. Everyone assumes that I'm straight and that's she's a friend it's really pissing me off.”

Meanwhile a non-binary queer bisexual younger person (25–34 year old age group) spoke of modifying their gender identity when undertaking a care placement which involved working with people living with dementia:

There's definitely times where I have to be in a closet still and it feels like that's the safest way to go about it. So I've just finished my placement for year one, so it's 120 h. I really, really enjoyed it but I had to pretend to be cis the whole time because it was an older adults' residential home where most of them have dementia and just trying to be non-binary in that context would be very, very complicated, I feel, so I just didn't approach it, basically. And I didn't ever talk about being queer or anything like that, but my boss did make some comment about Pride that implies that he knows that I'm queer anyway. So, apparently, I'm not very good at looking straight, which is fine (laughter).

We can read the above interview extract as both normative and queer. On the one hand, this non-binary bisexual younger person feels that it is important to “hide” their identity from older people with dementia because “in that context (it) would be very, very complicated.” On the other hand, they wonder if their queer identity was really “hidden,” since their manager appeared to notice, but no mention is made whether residents with dementia considered this too. It did not occur to this individual that dementia itself might queer questions of gender identity or indeed that those being cared for could be queer themselves. Hence, a queer(y)ing of cognitive difference in aging does recognize this, but also shows how manifest and impactful it can be. Indeed, without wishing to accuse or blame this particular individual, since they are subject to cisheteronormativity themselves, there is a sense in which these norms in relation to dementia remain frustratingly intact.

Meanwhile, other participants emphasized how queerness and non-familial relationality could be actively used to form supportive relationships to care for those living with dementia. This seemed to be the case for older participants who had first hand experience. In a discussion with an older cisgender gay man (75–84 year age group), when asked about getting older, he stated:

Well, I took it in my stride, and in the same way that I'd started to organize things with dementia care, it seemed to those of us who were working in that sphere with the (removed place) that the move from looking at the needs of people caring for someone with dementia and looking at people who were getting older was a very small step, and we made a conscious decision that we would be involved in discussing and promulgating the needs of older LGBT.

This middle-class man had experience of caring for a partner who had been diagnosed with dementia and had subsequently become involved in dementia activism and that related to older LGBTQ+ people's rights. He had considerable supportive networks to draw upon. During the interview he made several references to the importance of friendship, informal caring and non-familial relationality as forms of support for him. Indeed, this is very similar in terms of Halberstam's (2011) point about queer relationality and its possibilities – since cisheteronormativity often means queers are positioned in caring roles for others, they have, in effect, had to find alternative ways to manage beyond a cisheteronormative framework. Despite this, the overwhelming framing of dementia in the CILIA project interviews highlighted the disciplinary power of cisheteronormativity and cognonormativity that in many ways positioned queers living with dementia within oppressive and normative ontologies and structures. Yet as indicated above not all LGBTQ+ individuals will have the social, cultural and indeed financial resources to alleviate this.

Thus, far we have considered ways in which Queer Theory can highlight and challenge ways we think about aspects of aging related to time and cognition. Queer Theory reminds us of the messy compromises and normative framings that shape and indeed constrain LGBTQ+ people's experiences of aging. It highlights why we need to think critically about how LGBTQ+ people are positioned by institutions and services as they get older and how they can and cannot respond. In the final subsection, we will discuss issues of frailty and vulnerability that are frequently associated with aging, older people and later life. In so doing, we want to further show the usefulness of combining queer theoretical discussions with some empirical examples.

### Queering frailty and vulnerability

The concept of frailty is complex and often includes objective measures of age-related comorbidities that have an impact on health and wellbeing, as well as subjective feelings of fragility and vulnerability with negative cultural associations that are often stereotypical and ageist (Durepos et al., 2022). To be labeled as frail, either by oneself or by others is another marker of difference from norms associated with health, success and wisdom; it has become associated with a “fourth age” in a social imagination that posits it as a period of later life associated with decline, vulnerability and death (Gilleard and Higgs, 2011).

As both Pickard (2018) and Grenier (2020) have noted, frailty is mostly framed in biomedical terms which ignores not only individual perceptions and understandings, but crucially the political and economic contexts in which it is manifested. It is not our intention here to provide an in-depth discussion of this debate, nor to offer an extended discussion of frailty and vulnerability, but to consider how Queer Theory can trouble issues associated with frailty and vulnerability in LGBTQ+ people's lives and their understandings of aging, which can contribute toward a queer(y)ing of aging more broadly.

In this section, as well as drawing on the data collected in the CILIA project, we also draw more heavily on data from the earlier SAFE Housing project, in which these issues emerged during focus groups on housing in later life. In both projects, frailty and vulnerability were conjoined and linked to aging, often in relation to physical and mental changes associated with later life. However, what made these points particularly *queer* was a linking to LGBTQ+ identities and experiences and, as significantly, to wider legal, political and cultural contexts. In short, frailty and vulnerability were *queered* because queers experience them differently and explain them in those terms.

Two authors who have written extensively about the politics of vulnerability and frailty and how this impacts the lives of specific groups of people, including LGBTQ+ people are Butler (2004a) and Puar (2017). Whilst the former is heavily associated with Queer Theory, Puar engages more critically with Queer Theory and LGBTQ+ scholarship, often in relation to critical race and decolonial theories. Both authors, despite using different examples, point us toward the politics of liveability; that is, how some lives are more precarious and how institutions actively and purposively make some lives frail and vulnerable, a process Puar's (2017) refers to as debilitation.

In both the CILIA and SAFE Housing projects, it was often the disciplinary effects of institutions and institutional subjects (people) that were cited by participants when discussion turned to both frailty and vulnerability. In these cases, participants made being LGBTQ+ as relevant to how these might be or were experienced. They draw attention toward the power of cisheteronormativity to discipline their aging lives, making them vulnerable and exacerbating frailty.

Whilst decisions to reveal one's gender identity and sexual orientation to others or not are identified frequently in the literature on sexuality and aging (King, 2016a), a link between this with frailty and an increasing vulnerability were cited by participants as important. One cisgender lesbian (55–64 year age group) summarized this point during a focus group, when she emphasized the cumulative and affective toll on the self; or as she phrased it, the “exhaustion of constantly coming out,” making a link between this and being more frail:

just all the little things, and we've talked around this a lot (in the focus group) haven't we, all of us probably, but just the constant exhaustion, if you were feeling frail, you were very old, you are perhaps ill, the exhaustion of constantly coming out or not, or even thinking “no I'm not going to come out,” you know, should I or shouldn't I?

This was also emphasized by another focus group participant, not in terms of an affective impact on self, but rather in social and relational terms on the affect of others. As this 67-year old, cisgender lesbian, explained:

But I mean one might feel a lot more frail, a lot more vulnerable, so that when you're that age you don't want to be fielding it in the way that you've fielded it all your life and being circumspect and watching what pronouns you use and, in order to, let's face it, spare somebody else's feelings most of the time isn't it? To try and protect them isn't it?

As such, this woman identifies how even in later life, when, as she puts it, “one might feel a lot more frail, a lot more vulnerable,” identity management remains for her a pertinent practice. Reading this through a queer theoretical lens indicates the power of cisheteronormativity on the ontological bearing of some LGBTQ+ people as they age, as they experience this aspect of aging; it is a queer(y)ing of aging because it is not something that cisgender and heterosexual people do – vulnerability and frailty are not exacerbated by their gender identity and sexual orientation. Indeed, a non-binary bisexual person (age group 45–54) interviewed in the CILIA project spoke of the cumulative impact of cisheteronormativity on their health and how, they believed, that could make them “decrepit” later in life. They explained this in relation to GP consultations and not seeking medical advice:

When you're in a consultation with a GP, you are a weak and vulnerable person and they have all the power because, you know, the nature of the space that you're in, the situation that you're in. And things like that make you go, “Oh look, there's this, it'll probably clear up. I won't go, it'll just be grief.” And that means that over time you build up, bigger health problems than your cis friends do, than your straight friends do, and that makes me think that in later life, you know, I am likely to be a decrepit old person rather than a healthy old person type of thing, that there are more likely to be more things that build up.

The point made by this participant indicates how, for them, interactions with institutions, particularly those deemed to be cisheteronormative, may have longer term consequences for health and wellbeing later in life. The decision about whether to go to the GP or not is related specifically to the “grief” it may cause at the time and the longer terms health consequences because of their own trans identity. It does not, of course, mean that this will happen, or indeed that it will happen to all LGBTQ+ people, but there is always a potential for institutionalized cisheteronormativity, as a structural form of inequality, to provoke this. In this respect, this represents a process of debilitation (Puar, 2017) for queer people; they are made systemically and institutionally frail as a result.

One arena in which possibilities for frailty and vulnerability to be exacerbated by cisheteronormativity, and thereby where a process of debilitation can be magnified, is in housing. As noted above, the SAFE Housing project specifically researched housing, although it was also referred to by participants in CILIA. There is a growing literature about older LGBT+ people's concerns about specialist housing and care for older people. This illustrates the cisheteronormativity of these contexts (Westwood, 2016b; Hafford-Letchfield et al., 2018; Willis et al., 2018; Almack et al., 2022), including the effects of LGBTQ+ hypervigilance around housing and its impact on mental health (Stoneman et al., 2020). However, considering these contexts as creating frailty and vulnerability as a process of debilitation in queer theoretical terms, has not been considered before in a sociology of aging.

Participants in both studies highlighted different types of housing and care that could invoke this process and how they would try to remain vigilant to it. For instance, one older cisgender lesbian (65–74 year age group) in the CILIA project emphasized how she wanted to stay healthy, not simply for reasons of wellbeing, but directly to avoid discrimination in residential or nursing home care settings. As she said:

I want to stay healthy. I think probably staying healthy and independent is the single most important thing because you become obviously very vulnerable and you start to rely on external service. And one thing I do know, and it does please me, is that only about 3% of the older generation are in nursing homes or residential homes but that would be a living hell for me because that's where I'm going to be faced with homophobia, and so that's my only fear.

Many of the cisgender gay men in the SAFE Housing project talked about vulnerability when being cared for by those who entered their home e.g., domiciliary carers. There was a strong preference for carers of the same sexual orientation as themselves. Meanwhile, another older cisgender lesbian (in the 65–74 year old age group) said during one of the SAFE Housing focus groups that she was not concerned about domiciliary care providers seeing signifiers of her sexuality in items around her home. However, she emphasized how that may change if she became “much frailer”:

I'm not bothered about that because I don't care who comes in my home. I've got everything that would indicate that I'm not straight (laughs). Pictures, pictures of me at Pride, lots of different things, books, you know, masses and masses of books. Yeah there's lots of things like that and I think, I know that that bothers some people because I know people that are frailer than me that have to hide their stuff away in case somebody, a cleaner or somebody, sees it. I don't, you know, have to do any of that. It may be different when I get, you know, if I live long enough and I'm in my nineties or something and I'm much frailer, it may be different, maybe I might feel more vulnerable.

These participants highlight not only their individual concerns, but a recognition that frailty and vulnerability can be intrinsically linked to cisheteronormativity and the discrimination and prejudice that follows from it for LGBTQ+ people who require institutional care. They link the personal and interactional level of their own lives with wider structural constraints pertaining to gender identity and sexuality. Hence, to refer back to the previous point, it is not that LGBTQ+ people are frailer and more vulnerable as they get older compared to cisgender heterosexual people, but that cisheteronormativity largely channeled in and through institutions, creates a process of debilitation that queer people are mindful of and take steps, as far as possible, to resist. Whilst financial advantages may insulate against these, to an extent, even middle-class participants such as the woman quoted above, recognized that this was partial. Hence frailty and vulnerability, as ontological states that have be applied to a sociology of aging and critiqued as such, must also to be queered.

## Discussion and conclusion

In this article we have examined three aspects of aging by combining Queer Theory with empirical examples drawn from studies we have undertaken with LGBTQ+ people. We have undertaken this to add to the literature that *queers* aging and to further a queer sociology of aging. In doing so, we have highlighted the ways that normativities shape and constrain aging lives and especially the later lives of LGBTQ+ people. We have shown the usefulness of Queer Theory, as a theoretical framing, which has a range of conceptual tools that can be used to highlight and critically engage with the way that cisheteronormativity and cognonormativity are related to aging in terms of chronology, cognition and frailty and vulnerability. By grounding this in examples of data, we have also used it to show how older LGBTQ+ people themselves respond to, incorporate and sometimes challenge these normativities and we have also pointed out that not everyone can respond in the same way. In effect, we have taken the more esoteric and broad-brush concepts developed by Queer Theorists and applied them to examples of everyday lives.

As we have demonstrated, there is a messiness in people's responses to questions of chronology, cognition and frailty/vulnerability that belies simplistic binaries of well/unwell, healthy/ill, successful/unsuccessful that are often applied to aging. We have thereby extended more general Queer Theorical critiques applied to successful aging (Sandberg and Marshall, 2017) or applied to specific instances or identities (Fabbre, 2015). For us, Queer Theory highlights that messiness and complexity, but it also illuminates what, for LGBTQ+ people, are the underlying norms that anchor these and frequently normalize them. In doing so, we have achieved the key objective for this article. However, it would be remiss if we did not point out and consider the complications and limitations with such an endeavor, particularly problems with Queer Theory and how we have applied it here.

Whilst Queer Theory is useful to think through the constraining aspects of norms on people's lives, its largely deconstructionist approach toward identity can be problematic. In this sense, it has a tendency to overlook everyday interactions and experiences (Jackson and Scott, 2010), albeit ones shaped by social norms. However, as we have demonstrated in this article, there is no reason why a queer(y)ing of aging cannot combine insights from Queer Theory with everyday empirical examples. Indeed, it highlights further points raised by others, that Queer Theory can have a sociological orientation and application (Plummer, 2011; McCann, 2016), but in this case applied to aging, in ways that others have asserted in relation to allied disciplines such as gerontology, social work and public health (Ramirez-Valles, 2016; Fabbre, 2017; Sandberg and King, 2019).

A second area of concern in relation to Queer Theory's deconstructionist tendencies is, somewhat ironically, the occlusion of difference. Queer Theory often takes gender identity and/or sexuality as *the* markers of difference. Indeed this can be traced back to some of the earliest works of Queer Theory (Fuss, 1991; Sedgwick, 1993). In this respect, other intersectional forms of social division, oppression and empowerment, can be marginalized (Sandberg and King, 2019). There is, however, no reason why a more grounded empirical analysis of aging experiences, cannot combine a queer perspective with a more sociological and intersectional one. For instance, although in this article we have not disaggregated the multiple intersections applicable to the examples we have included in a systematic way, this does not mean such an analysis cannot be achieved. We have highlighted the intersection between gender identity, sexuality and age, and where appropriate social class. Elsewhere we have utilized intersectionality in relation to a specific area, LGBTQ+ employment (Hall et al., 2022) as well as differences in aging as a lesbian, gay or bisexual person (King, 2016a) as a way of examining intercategorical differences related to other identities. We have not done so here, because our focus has been on the applicability of Queer Theory *per se*, as an approach for critically engaging and disturbing norms associated with three aspects of aging to further elaborate a queer sociology of aging. However, we are mindful that further work could be undertaken to overcome some of the limitations with Queer Theory by synthesizing it with intersectionality more much more extensively.

Within the empirical examples we have discussed the body and embodiment in aging are apparent: frail and immobile bodies that are allegedly vulnerable, older brains that may lack cognitive capacity, for instance. Yet as other authors have noted, Queer Theory can be accused of downplaying the body or, conversely, downplaying or failing to fully address the symbolic violence that is often applied to bodies that are pathologized or marginalized – whether on the grounds of disability (Ramlow, 2009; Johnson, 2015), race (Muñoz, 1999; Moussawi and Vidal-Ortiz, 2020) or class (McDermott, 2011). We also recognize this critique. Certainly, the sociology and social gerontology of aging has much to offer Queer Theory in this respect. It can highlight, as we have done in this article how norms related to aging shape everyday lives, linking the discursive with the embodied through detailed empirical examples.

One principle ramification of the theoretical task we have undertaken in this article is to further consider the myriad ways that cisheteronormativity shapes and constrains the later lives of LGBTQ+ people and indeed all older people. As we noted in relation to frailty and vulnerability, Puar's (2017) ideas about debilitation and maiming could be particularly productive concepts to explore further. Arguably, cisheteronormativity can make LGBTQ+ older people a “population at risk” of debilitation because the institutions through which their lives are lived are biopolitical ones that have the potential to maim them. In short, institutional cisheteronormativity creates the conditions that frame LGBTQ+ aging and through which many of the health concerns expressed about LGBTQ+ populations are funneled. Whilst we think that a minority stress model (Savage and Barringer, 2021) can be useful in thinking about older LGBTQ+ lives in this way, it can individualize wider systemic, cultural and biopolitical norms and indeed sociological structures and forms of division highlighted within the discipline through the work of Bourdieu (1984, 1989). As such, the theoretical ideas we have outlined in this article provide an important adjunct because they are inherently political and critical, having a relentless focus on the power of norms to shape and constrain lives, but also how such norms are unstable and open to challenge and reorientation. It is for these reasons that we conclude that queer(y)ing aging is an important step in sociological studies of later life and the in/equalities that impact older people.

## Data availability statement

The datasets presented in this article are not readily available because of issues of confidentiality. Requests to access the datasets should be directed to AK.

## Ethics statement

The studies involving humans were approved by the University of Surrey Ethics Committee. The studies were conducted in accordance with the local legislation and institutional requirements. The participants provided their written informed consent to participate in this study.

## Author contributions

AK led the writing of this article, with critical insights from MH. MH led the analysis of the data. Both authors contributed to the article and approved the submitted version.

## Footnotes

^1^Because of the ethical and research governance approval processes, each national institution in the CILIA project has full ownership of the data collected in their own country but pooled data has only been used for full-project, co-authored publications. We are therefore not able to conduct a comparative analysis for this particular article.

## References

[B1] Almack K. King A. Jones R. L. (2022). Care in late life, end of life and in bereavement for the oldest LGBT generations around the globe. Sexualities 25, 3–8. 10.1177/1363460720986911

[B2] Bailey D. Calasanti T. Crowe A. di Lorito C. Hogan P. de Vries B.. (2022). Equal but different! Improving care for older LGBT+ adults. Age Ageing 51, 42. 10.1093/ageing/afac142 35751872

[B3] Baril A. Silverman M. (2019). Forgotten lives: trans older adults living with dementia at the intersection of cisgenderism, ableism/cogniticism and ageism. Sexualities 1363460719876835. 10.1177/1363460719876835

[B4] Bourdieu P. (1984). Distinction: A Social Critique of the Judgement of Taste. London: Routledge and Keegan Paul.

[B5] Bourdieu P. (1989). Social space and symbolic power. Sociol. Theor. 7, 14–25. 10.2307/202060

[B6] Boyatzis R. E. (1998). Transforming Qualitative Information: Thematic Analysis and Code Development. London: Sage Publications.

[B7] Butler J. (1993). Bodies that Matter: On the Discursive Limits of Sex. New York: Routledge.

[B8] Butler J. (2004a). Precarious Life. London: Verso.

[B9] Butler J. (2004b). Undoing Gender. London: Routledge.

[B10] Cronin A. (2006). “Sexuality in gerontology: a heteronormative presence, a queer absence,” in Ageing and Diversity: Multiple pathways and cultural migrations, eds. Daatland S.O. Biggs S. (Bristol: Policy Press),107–122.

[B11] Cronin A. (2008). “Focus Groups,” in Researching Social Life, ed. Gilbert G.N. (London: Sage), 224–244.

[B12] Di Lorito T. Bosco C. Peel A. Hinchliff E. Dening S. Calasanti T.. (2022). Are dementia services and support organisations meeting the needs of lesbian, gay, bisexual and transgender (LGBT) caregivers of LGBT people living with dementia? A scoping review of the literature. Aging Mental Health 26, 1912–1921. 10.1080/13607863.2021.2008870 34842010

[B13] Durepos P. Sakamoto M. Alsbury K. Hewston P. Borges J. Takaoka A.. (2022). Older adults' perceptions of frailty language: a scoping review. Can. J. Aging 41, 193–202. 10.1017/S0714980821000180 34253271

[B14] Edelman L. (2004). No Future: Queer Theory and the Death Drive. Durham, NC: Duke University Press.

[B15] Fabbre V. (2017). Queer aging: implications for social work practice with lesbian, gay, bisexual, transgender, and queer older adults. Soc. Work 62, 73–76. 10.1093/sw/sww076 28395042

[B16] Fabbre V. D. (2015). Gender transitions in later life: a queer perspective on successful aging. The Gerontologist 55, 144–153. 10.1093/geront/gnu079 25161264 PMC4542896

[B17] Formby E. (2017). Exploring LGBT Spaces and Communities: Contrasting Identities, Belongings and Wellbeing. London: Routledge.

[B18] Foucault M. (1978). The Will to Knowledge - The History of Sexuality, Vol. 1. London: Penguin.

[B19] Freeman E. (2010). Time Binds: Queer Temporalities, Queer Histories. Durham, NC: Duke University Press.

[B20] Fuss D. (1991). “Inside/Out,” in Inside/Out: Lesbian Theories, Gay Theories, ed. Fuss D. (London: Routledge), 1–10.

[B21] Gilleard C. Higgs P. (2011). Ageing abjection and embodiment in the fourth age. J. Aging Stu. 25, 135–142. 10.1016/j.jaging.2010.08.018

[B22] Green A. I. (2007). Queer theory and sociology: locating the subject and the self in sexuality studies. Sociol. Theor. 25, 26–45. 10.1111/j.1467-9558.2007.00296.x

[B23] Grenier A. M. (2020). The conspicuous absence of the social, emotional and political aspects of frailty: the example of the white book on frailty. Ageing Soc. 40, 2338–2354. 10.1017/S0144686X19000631

[B24] Hafford-Letchfield T. Simpson P. Willis P. B. Almack K. (2018). Developing inclusive residential care for older lesbian, gay, bisexual and trans (LGBT) people: an evaluation of the care home challenge action research project. Health Soc. Care Commun. 26, e312–e320. 10.1111/hsc.12521 29181893

[B25] Halberstam J. (2005). In a Queer Time and Place: Transgender Bodies, Subcultural Lives. New York: NYU Press.

[B26] Halberstam J. (2011). The Queer Art of Failure. Durham: Duke University Press.

[B27] Hall M. A. Barbrook-Johnson P. Bayrakdar S. King A. (2022). Queer(y)ing agent-based modeling for use in LGBTQ studies: an example from workplace inequalities. J Homosex. 5, 1–27. 10.1080/00918369.2022.2106464 35984389

[B28] Jackson S. Scott S. (2010). Theorising Sexuality. Buckingham: Open University Press.

[B29] Johnson M. L. (2015). Bad romance: a crip feminist critique of queer failure. Hypatia 30, 251–267. 10.1111/hypa.12134

[B30] King A. (2016a). Older Lesbian, Gay and Bisexual Adults: Identities, Intersections and Institutions. London: Routledge.

[B31] King A. (2016b). “Queer(y)ing dementia – bringing queer theory and studies of dementia into dialogue,” in Lesbian, Gay, Bisexual and Transgender (LGBT) Individuals Living with Dementia, eds. Price E. Westwood S. (London: Routledge).

[B32] King A. (2022). Queer futures? Forget it! Dementia, queer theory and the limits of normativity. J. Aging Stu. 63, 1–8. 10.1016/j.jaging.2021.100993 36462926

[B33] King A. Cronin A. (2010). “Queer Methods and Queer Practices: Re-examining the identities of older lesbian, gay, bisexual (OLGB) adults,” in Queer Methods and Methodologies, eds. Browne K. Nash C. (Aldershot: Ashgate) 85–96.

[B34] Lahire B. (2011). The Plural Actor. Cambridge: Polity.

[B35] McCann H. (2016). Epistemology of the subject: queer theory's challenge to feminist sociology. Women's Stu. Q. 44, 224–243. 10.1353/wsq.2016.0044

[B36] McCann H. Monaghan W. (2020). Queer Theory Now: From Foundations to Futures. London: Red Globe Press.

[B37] McDermott E. (2011). The world some have won: sexuality, class and inequality. Sexualities 14, 63–78. 10.1177/1363460710390566

[B38] McDermott E. Hughes E. Rawlings V. (2017). The social determinants of lesbian, gay, bisexual and transgender youth suicidality in England: a mixed methods study. J. Pub. Health 40, e244–e251. 10.1093/pubmed/fdx135 29045707 PMC6166581

[B39] Moussawi G. Vidal-Ortiz S. (2020). A queer sociology: on power, race, and decentering whiteness. Sociol. Forum 35, 1272–1289. 10.1111/socf.12647

[B40] Muñoz J. E. (1999). Disidentifications: Queers of color and the performance of politics. Minneapolis, MN: University of Minnesota Press.

[B41] Pickard S. (2018). Health, illness and frailty in old age: a phenomenological exploration. J. Aging Stu. 47, 24–31. 10.1016/j.jaging.2018.10.002 30447866

[B42] Plummer K. (2011). “Critical humanism and queer theory: living with the tensions,” in The SAGE Handbook of Qualitative Research, eds. Denzin N.K. Lincoln Y.S. (London: Sage), 195–207.

[B43] Puar J. (2017). The Right to Maim: Debility, Capacity, Disability. Durham, NC: Duke University Press.

[B44] Pugh S. (2002). “The forgotten: a community without a generation - older lesbian and gay men,” in Handbook of Lesbian and Gay Studies, eds. Richardson D. Seidman S. (London: Sage Publications), 161–182.

[B45] Ramirez-Valles J. (2016). Queer Aging: The Gay by Boomers and a New Frontier for Gerontology. Oxford: Oxford University Press.

[B46] Ramlow T. R. (2009). “Queering, cripping,” in The Ashgate Companion to Queer Theory, eds. Giffney N. O'Rourke M. (Farnham: Ashgate), 129–146.

[B47] Rosenfeld D. (2002). “Identity careers of older gay men and lesbians,” in Ways of Aging, eds. Gubrium F. Holstein J. (Oxford: Blackwell), 160–181.

[B48] Sandberg L. King A. (2019). “Queering gerontology,” in Encyclopedia of Gerontology and Population Aging (Living Edition), ed Danan G. (Cham: Springer International Publishing), 4108–4144.

[B49] Sandberg L. Marshall B. (2017). Queering aging futures. Societies 7, 1–11. 10.3390/soc7030021

[B50] Savage B. Barringer M. N. (2021). The (minority) stress of hiding: the effects of LGBT identities and social support on aging adults' concern about housing. Sociol. Spectr. 41, 478–498. 10.1080/02732173.2021.2010627

[B51] Seal A. (2016). “Thematic Analysis,” in Researching Social Life, eds. Gilbert G.N. Stoneman P.. *4th ed* (London: Sage) 443–460.

[B52] Sedgwick E. K. (1993). “Epistemology of the closet,” in The Lesbian and Gay Studies Reader, eds. Abelove H. Barale M.A. Halperin D.M. (New York, NY: Routledge), 45–61.

[B53] Smith L. Chesher I. Fredriksen-Goldsen K. Ward R. Phillipson L. Newman C. E.. (2022). Investigating the lived experience of LGBT+ people with dementia and their care partners: a scoping review. Ageing Soc. 14, 1–24. 10.1017/S0144686X22000538

[B54] Smith M. (2022). Where Does the British Public Stand on Transgender Rights in 2022? London: YouGuv.

[B55] Stoneman P. Allum N. Hazeldine N. King A. (2020). Vigilance Within the LGBTQ+ *Community and Its Effect on Mental Wellbeing*. Available online at: https://ssrn.com/abstract=3745320

[B56] Sullivan N. (2003). A Critical Introduction to Queer Theory. Edinburgh: Edinburgh University Press.

[B57] Taylor Y. Hines S. Casey M. E. (2010). Theorizing Intersectionality and Sexuality. London: Palgrave MacMillan.

[B58] Traies J. (2016). The Lives of Older Lesbians: Sexuality, Identity and the Life Course. London: Palgrave MacMillan.

[B59] West C. Zimmerman D. H. (1987). Doing gender. Gender Soc. 1, 125–151. 10.1177/0891243287001002002

[B60] Westwood S. (2016a). Ageing, Gender and Sexuality: Equality in Later Life. London: Routledge.

[B61] Westwood S. (2016b). ‘We see it as being heterosexualised, being put into a care home': gender, sexuality and housing/care preferences among older LGB individuals in the UK. Health Soc. Care Commun. 24, 155–163. 10.1111/hsc.12265 26304109

[B62] Willis P. Raithby M. Maegusuku-Hewett T. (2018). “Fabled and far-off places: the preferred futures of older lesbian and gay adults in long-term care environments,” in Older Lesbian, Gay, Bisexual and Trans People: Minding the Knowledge Gaps, eds. King A. Almack K. Suen Y.T. Westwood S.. (London: Routledge), 142–157.

[B63] World Health Organization (2021). Dementia. Geneva: World Health Organization.

